# Familial Mediterranean Fever with Neonatal Onset: Case Report

**DOI:** 10.1155/2020/6649525

**Published:** 2020-12-14

**Authors:** Kübra Arslan, Serdar Ümit Sarici, Gonca Kolukisa, Demet Altun

**Affiliations:** Department of Pediatrics, Ufuk University Faculty of Medicine, Ankara, Turkey

## Abstract

Familial Mediterranean fever (FMF) is an autosomal recessively inherited disorder characterized by recurrent fever and attacks of abdominal pain, chest pain, and joint pain. Attacks of recurrent fever and serositis are encountered clinically. Attacks may present either with only one symptom or many simultaneous symptoms. Although most of the patients are diagnosed clinically above the age of 2, those cases who are diagnosed before 2 years of age and with clinical course of isolated fever are believed to have a more serious course and tend to develop amyloidosis. In this article, a case who was admitted first on the 22nd day of life and later diagnosed to have FMF with recurrent attacks of isolated fever and no other focus is presented. We emphasize that FMF may present as early as in the first month of life, and it should be considered in cases presenting with fever of unknown origin and misdiagnosed to have late neonatal sepsis or occult bacteremia at this age group.

## 1. Introduction

Familial Mediterranean fever (FMF) is an autosomal recessively inherited disorder caused by genetically inherited mutations [1]. It is seen particularly in Jews, Armenians, Turks, Arabs, and in other ethnic groups living in the Mediterranean region [2]. Clinical symptoms are fever, abdominal, chest, or joint pain recurring with irregular intervals, erythema like erysipelas in the lower extremities, myalgia, orchitis, and aseptic meningitis. The common characteristic of these symptoms is unresponsiveness to analgesics or antipyretics and complete resolution spontaneously in 24 to 72 hours [1]. Course of attack may be with only one symptom or multiple symptoms [3]. These symptoms appear before 20 years of age in more than 90% of cases, and in 60% of cases, the age of onset is less than 10 years of age [4]. Symptoms related to FMF are frequently and easily detected when the child reaches the age at which he or she expresses his or her own complaints clearly. Thus, the number of cases diagnosed before the age of two is very low although the symptoms of the disease are very clear [5].

In this article, a 6-month-old male patient who was first admitted with fever and positive laboratory findings of leukocytosis and high CRP on day 22 of life diagnosed to have and treated for late neonatal sepsis and later was diagnosed to have FMF with recurrent attacks of fever at 6 months of life is presented. We thus emphasize that FMF may present as early as in the neonatal period, some newborns diagnosed to have late neonatal sepsis or occult bacteremia might have been misdiagnosed indeed, and signs and symptoms of neonatal sepsis should also alert the attending physicians with respect to this diagnosis, particularly if the family history is positive.

## 2. Case Presentation

A 71-day-old male patient was admitted with a complaint of fever. In the patient history, he had been hospitalized and treated for neonatal sepsis due to fever with no focus at another center on day 22 of life. In the family history, he had an elder sister with an age of 11 with the diagnosis of FMF. Physical examination of the patient was thoroughly normal, and the laboratory results were white blood cells (WBCs): 22300/mm^3^, hemoglobin (Hb): 10.4 g/dL, thrombocytes: 457000/mm^3^, and C-reactive protein (CRP): 39.3 mg/L (0–5 mg/L). Ceftriaxone was started intravenously towards the possible diagnosis of occult bacteremia and complications of late neonatal sepsis. After 72 hours of treatment and a fever-free period of 48 hours, the patient was discharged. Four months later, at the age of 5.5 months, the patient was admitted with the complaint of fever. In physical examination, there was no abnormality and no focus of fever. Laboratory investigation showed WBCs: 17000/mm^3^, Hb: 9.56 g/dL, thrombocytes: 504000/mm^3^, erythrocyte sedimentation rate (ESR): 29 mm/h, and CRP: 73.8 mg/L (0–5 mg/L). The patient was discharged provided that he would be followed in an outpatient manner. Fifteen days later, the patient was again admitted with the complaint of isolated fever, and results of the laboratory investigation were as follows: WBCs: 17200/mm^3^, Hb: 9.03 g/dL, thrombocytes: 520000/mm^3^, CRP: 208 mg/L (0–5 mg/L), and serum amyloid A (SAA): 949 mg/L (0–7 mg/L). Gene mutation analysis (MEFV) was ordered considering recurrent attacks of fever with no focus, high acute phase reactants during the attacks, highly increased level of SAA, and the history of elder sister with FMF, and the result was found as homozygous positive with respect to M694V.

In presenting the case, both verbal and written informed consent was obtained from the parents.

## 3. Discussion

In this article, a male infant who was followed due to recurrent isolated fever attacks between the late neonatal period and 6 months of age and finally diagnosed to have FMF at the end of this period is presented. Although FMF is a disease which is most frequently diagnosed during childhood, it may give symptoms in earlier ages. In a study comparing patients diagnosed before and after 3 years of age, mean Hb and WBC levels were reportedly lower and higher, respectively, in patients diagnosed before 3 years of age [6]. In addition, smaller children were more prone to have higher acute phase reactant (APR) levels [6], and to have more serious attacks, with an increased risk of amyloidosis, and the need of higher colchicine doses in the follow-up [6, 7]. Our patient also had higher WBC and APR levels during all of the three attacks we observed.

The most frequently observed symptoms of FMF in children are fever and abdominal pain, and however, some clinical, molecular, and prognostic characteristics may differ between patients with early and late (adulthood) onset [8]. In a study, 6.2% of children with FMF had attacks of isolated fever alone without any serositis [2]. The probability of attacks with isolated fever alone increases with decreasing age, and the frequency of arthritis and erysipelas like skin eruptions increases in patients with early onset of FMF [8]. Thus, unnecessary diagnostic and therapeutic interventions should be avoided in these patients considering the increased frequency of arthritis/arthralgia and skin eruptions in patients with early onset. The only symptoms of presentation in our case in all of the 3 attacks we observed were fever alone, and the diagnosis of FMF should be remembered in mind in smaller infants and even newborns presenting with fever alone and normal physical examination and laboratory findings. However, the most important risk is the possibility of skipping diagnosis of a real neonatal sepsis, microbiologically proven or not, and this is a major diagnostic limitation in establishing diagnosis of FMF in smaller infants.

In a retrospective study reporting 317 pediatric patients with FMF, 19 cases (6%) had symptoms in the first 30 days after birth [9]. Family history and homozygous M694V mutation were positive in 60% and 42% of patients, respectively [9]. Start of the disease was also in the first 30 days of life; family history and M694V mutation were also positive in our patient, resembling the findings of that study [9].

In another study about patients with pediatric FMF, the disease onset was earlier, attacks were shorter, family history was more positive, and homozygous M694V mutation was more frequent in patients with fever as the initial presenting symptom when compared to patients with serositis as the initial presenting symptom [2]. Our patient presenting as early as the 22nd day of life also had both positivities of family history and homozygous M694V mutation.

In conclusion, it should be remembered in mind that FMF may present as early as the 22nd day of life, as in our case, and the diagnosis of FMF should be considered in newborn infants and smaller infants presenting with attacks of isolated and recurrent fever and normal physical examination findings, especially if the family history is positive. However, this approach should not mislead the attending physicians with respect to skipping the diagnosis of a real neonatal sepsis when trying to make diagnosis of FMF in smaller infants.

## References

[1] Tufan A., Lachmann H. J. (2020). Familial Mediterranean fever, from pathogenesis to treatment: a contemporary review. *Turkish Journal of Medical Sciences*.

[2] Padeh S., Livneh A., Pras E. (2010). Familial Mediterranean fever in children presenting with attacks of fever alone. *The Journal of Rheumatology*.

[3] Maggio M. C., Corsello G. (2020). FMF is not always “fever”: from clinical presentation to “treat to target. *Italian Journal of Pediatrics*.

[4] Sohar E., Gafni J., Pras M., Heller H. (1967). Familial Mediterranean fever. *The American Journal of Medicine*.

[5] Keskindemirci G., Aktay Ayaz N., Aldemir E., Aydogmus C., Aydogan G., Kavuncuoglu S. (2014). Familial Mediterranean fever: diagnosing as early as 3 months of age. *Case Reports in Pediatrics*.

[6] Yalcinkaya F., Ozcakar Z. B., Tanyildiz M., Elhan A. H. (2011). Familial Mediterrenean fever in small children in Turkey. *Clinical and Experimental Rheumatology*.

[7] Özdel S., Özçakar Z. B., Kunt S. Ş., Elhan A. H., Yalçınkaya F. (2016). Late-onset disease is associated with a mild phenotype in children with familial Mediterranean fever. *Clinical Rheumatology*.

[8] Üreten K., Gönülalan G., Akbal E. (2010). Demographic, clinical and mutational characteristics of Turkish familial Mediterranean fever patients: results of a single center in Central Anatolia. *Rheumatology International*.

[9] Çelikel E., Özçakar Z. B., Özdel S. (2019). Neonatal onset familial Mediterranean fever. *Modern Rheumatology*.

